# Automatic renal mass segmentation and classification on CT images based on 3D U-Net and ResNet algorithms

**DOI:** 10.3389/fonc.2023.1169922

**Published:** 2023-05-18

**Authors:** Tongtong Zhao, Zhaonan Sun, Ying Guo, Yumeng Sun, Yaofeng Zhang, Xiaoying Wang

**Affiliations:** ^1^ Department of Radiology, Peking University First Hospital, Beijing, China; ^2^ Department of Development and Research, Beijing Smart Tree Medical Technology Co. Ltd., Beijing, China

**Keywords:** renal mass, contrast-enhanced computed tomography, deep learning, U-Net, residual network

## Abstract

**Purpose:**

To automatically evaluate renal masses in CT images by using a cascade 3D U-Net- and ResNet-based method to accurately segment and classify focal renal lesions.

**Material and Methods:**

We used an institutional dataset comprising 610 CT image series from 490 patients from August 2009 to August 2021 to train and evaluate the proposed method. We first determined the boundaries of the kidneys on the CT images utilizing a 3D U-Net-based method to be used as a region of interest to search for renal mass. An ensemble learning model based on 3D U-Net was then used to detect and segment the masses, followed by a ResNet algorithm for classification. Our algorithm was evaluated with an external validation dataset and kidney tumor segmentation (KiTS21) challenge dataset.

**Results:**

The algorithm achieved a Dice similarity coefficient (DSC) of 0.99 for bilateral kidney boundary segmentation in the test set. The average DSC for renal mass delineation using the 3D U-Net was 0.75 and 0.83. Our method detected renal masses with recalls of 84.54% and 75.90%. The classification accuracy in the test set was 86.05% for masses (<5 mm) and 91.97% for masses (≥5 mm).

**Conclusion:**

We developed a deep learning-based method for fully automated segmentation and classification of renal masses in CT images. Testing of this algorithm showed that it has the capability of accurately localizing and classifying renal masses.

## 1 Introduction

The detection of many renal masses is often accidental during abdominal CT imaging (1, 2). Although most renal masses are benign, such as simple cysts (2, 3), up to 70-80% of solid masses are malignant and often caused by renal cell carcinoma (RCC) (4). Proper characterization of these masses is crucial to ensure effective treatment planning, as they can be life-threatening issues (1).

The first step toward evaluation of a renal mass is to determine if it is cystic or solid (3). Radiologists can distinguish cystic from solid renal masses based on CT images with fairly high levels of accuracy, but it is still challenging to differentiate malignant from benign solid renal masses (5, 6). The fatty component within the lesion is conventionally thought to be essential for the diagnosis of angiomyolipoma (AML); however, many pathologically proven AMLs do not show fatty tissue on imaging, causing difficulties in diagnosis (7, 8). Preliminary studies evaluating quantitative CT radiomic features have revealed promising results for determining the nature of renal masses and predicting subtypes of RCC (9–11).

Convolutional neural network (CNN) is a proficient tool for image segmentation and classification. U-Net, a modified version of the fully convolutional network, has been used for medical image analysis across different organs and has previously shown high segmentation and localization accuracy (12–15). As a deep learning model, the residual network (ResNet) has also been applied successfully in the fields of text classification and image classification (16–19). To our knowledge, the use of automatic deep learning techniques for the detection and characterization of renal masses has been little studied. To achieve a fully automatic noninvasive diagnosis process in CT images, bilateral kidneys must be first located and segmented. Then, focal renal lesions should be accurately detected and segmented, and the nature of lesions can be determined.

Our study aimed to develop a robust and automated pipeline for renal mass segmentation and classification on CT images. To achieve this, we selected the U-Net and ResNet models for their specific strengths in medical image segmentation and classification tasks, respectively. By combining the strengths of both models, we were able to create a cascade U-Net- and ResNet-based method that accurately segmented and classified focal renal lesions. Overall, our goal was to provide an automated approach for evaluating renal masses in CT images that could aid in diagnosis and treatment planning.

## 2 Materials and methods

This retrospective study was approved by the institutional review board (IRB), and the requirement for informed consent was waived. All the data were collected and deidentified under the Health Insurance Portability and Accountability Act.

### 2.1 Patients and data acquisition

**Figure 1:**
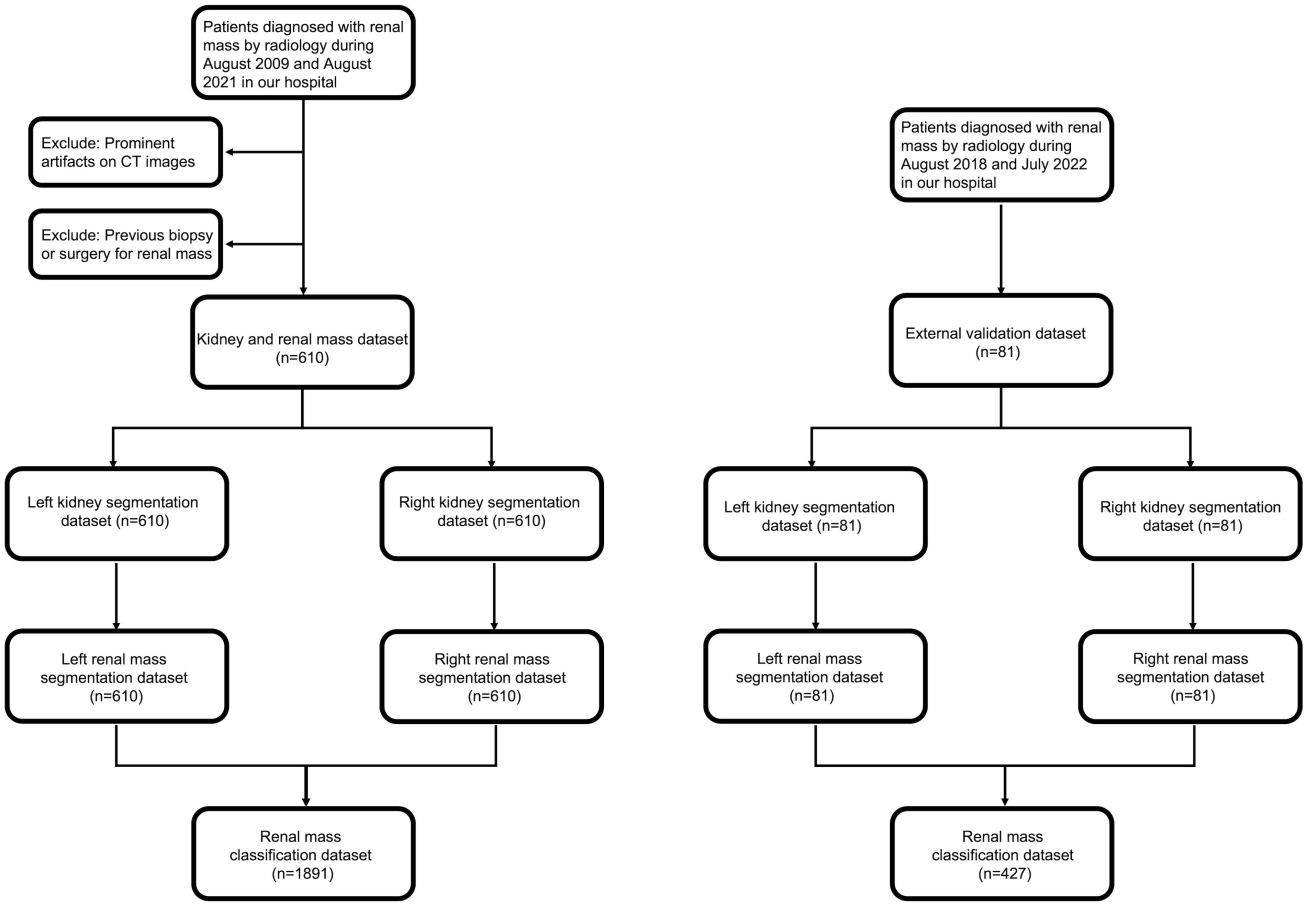
Flowchart of patient inclusion and exclusion.

This retrospective cohort study included patients with renal masses who underwent CT scans between August 2009 and July 2022 in our hospital (**Figure 1**). The commonly used inclusion criterion was contrast-enhanced CT images (corticomedullary and nephrographic phase, CMP and NP), and the exclusion criteria were as follows: (1) prominent artifacts on CT images and (2) previous biopsy or surgery for renal mass.

### 2.2 CT scan protocol

CT examinations were performed using a variety of multi-detector systems in our hospital (**Supplementary Table 1**), with all images available for review in our picture archiving and communication system (PACS). Patients were in the supine position, and contrast agent (iopromide 370 mgl/ml or iohexol 320 mgl/ml) was injected through the anterior elbow vein at a dose of 2 ml/kg and an injection flow rate of 2.5 ml/s.

All patients underwent four phases of CT scanning, including the plain scan, corticomedullary (30~35 s after contrast injection), nephrographic (60~70 s after contrast injection) and delay phase (190 ~ 200 s after contrast injection). The scanning tube voltage was 80~120 kV, the reconstructed slice thickness was 1~1.5 mm, and the reconstructed interval space was 1 mm.

### 2.3 Reference standard for CT image interpretation

Renal mass is defined as an abnormal growth in the kidney, excluding other conditions that may mimic a tumor, such as focal hypertrophy of the renal parenchyma, focal pyelonephritis, acute renal infarcts and renal pseudoaneurysms (20). After the detection of a renal mass, the first step in diagnosis is to differentiate a solid mass from a cystic mass, and this step mainly depends on enhancement criteria after injecting a contrast agent (21). According to the Bosniak classification (version 2019), we consider a cystic mass to be one in which less than approximately 25% of the mass is composed of enhancing tissue (3). Meanwhile, a mass consisting of wholly or mainly (> 25%) of tumor tissue with significant uptake of contrast (a change of more than 20 HU) is defined as solid (21). Once a renal mass has been cataloged as solid, the presence of macroscopic fat without calcification allows for the diagnosis of AML (22).

### 2.4 Manual segmentation

CT images in digital imaging and communication in medicine (DICOM) format were exported from PACS, and the images were transformed into neuroimaging informatics technology initiative (NIFTI) format for further investigation. The segmentation was performed with ITK-SNAP software (version 3.6.0) by a fellowship-trained radiologist with 15 years of experience in collaboration with a junior radiologist experienced in the analysis and segmentation of CT images.

**Figure 2:**
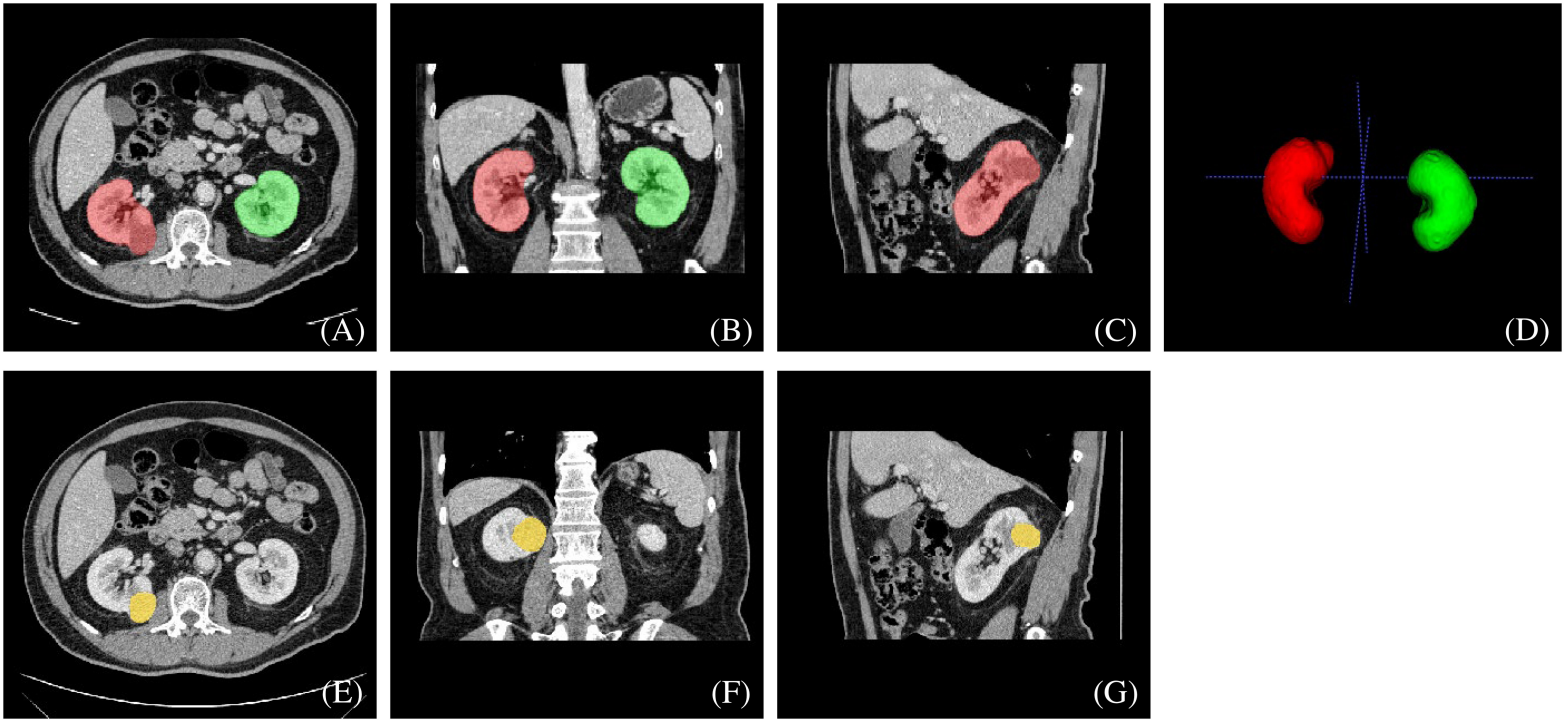
An example of manually segmented kidneys and renal mass from CT images. We used different mask colors to delineate different parts of the cross-section image: **(A–D)** red mask=right kidney, green mask=left kidney, **(E–G)** yellow mask=neoplasm.

The two radiologists then defined and manually segmented the boundaries of the kidneys, including the renal cortex, medulla and renal sinus, but excluded the retroperitoneal fat and hilar structures. The outline of the renal masses was delineated manually by the two radiologists. Contouring was drawn within the borders of the tumor masses, including necrotic, cystic change and hemorrhagic areas. For renal masses demonstrating an extension of the tumor into the renal veins or collecting system, only the mass proper was segmented. The two readers were blinded to the clinical and pathological information. An example of manual segmentation is presented in **Figure 2**.

### 2.5 Manual segmentation of KiTS21 dataset

The complete description of KiTS21 data can be found in (23). We utilized 300 labeled image series from the KiTS21 dataset to validate our proposed method for kidney and renal mass segmentation on CT images. Although there were some differences between our local dataset and the KiTS21 dataset, we were able to make adjustments to reconcile them. Specifically, in KiTS21, the renal sinus and kidney were identified as separate tissues, and the excess sinus fat included in the contour was automatically removed via a radiodensity threshold during postprocessing. Conversely, in our local dataset, everything within the margin of the outer boundary of the kidney, including the renal sinus, was labeled as being kidney. To achieve consistency in manual segmentation, two radiologists from our hospital modified the kidney labels in the KiTS21 dataset based on the criteria outlined in section 2.4. The renal mass labels in the KiTS21 dataset, on the other hand, were left unchanged.

### 2.6 Model training

The 610 image series were randomly assigned in an approximate ratio of 8:1:1 into the training, validation and test sets, and image series from the same patient were allocated to the same set. There were 487, 58 and 65 series in each subgroup, respectively. Before training, we adjusted the image window width to 30 HU and the window level to 300 HU.

**Figure 3:**
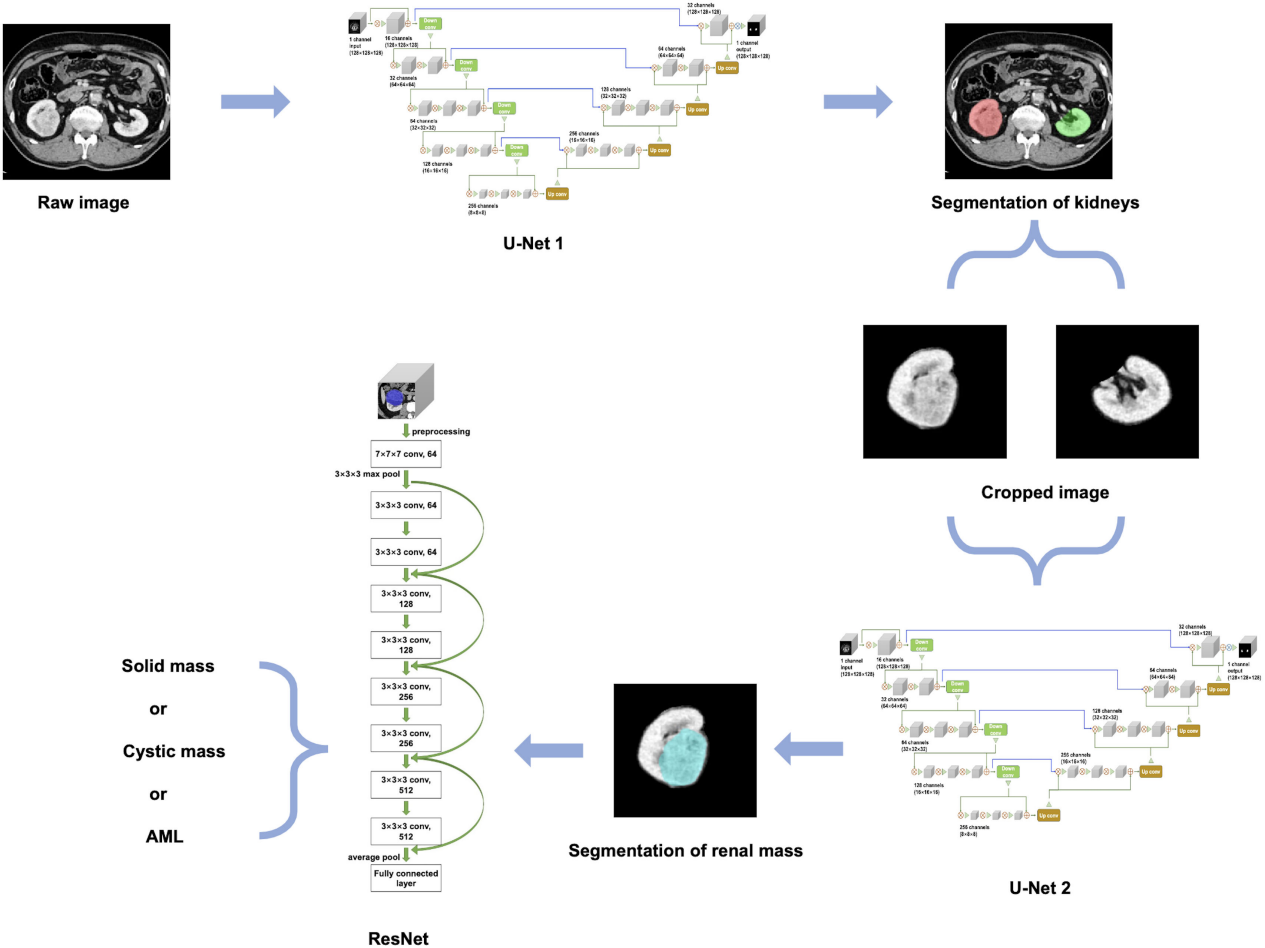
An overview of our proposed methodology.

An overview of our developed algorithm for renal mass segmentation and classification is shown in **Figure 3**.

#### 2.6.1 Segmentation model training

Before training the model, the images were resized from their original resolution of 512×512×N (N represents the number of layers in the image) to 128×128×128. Subsequently, various image augmentation methods were employed during model training, including random rotation (-10~10 degrees), random noise injection, and random horizontal and vertical translation (-0.1~0.1). This helped to increase the diversity of the dataset and prevent overfitting during model training.

The 3D U-Net architecture is a fully convolutional neural network that is specifically designed for volumetric segmentation tasks (24). It consists of an encoder-decoder structure with skip connections between them. The encoder pathway is designed to extract features from the input volume, while the decoder pathway is designed to reconstruct the output segmentation map from the encoded features. The skip connections help to preserve the spatial information and improve the accuracy of the segmentation. To provide a detailed description of the network architectures used in our study, we have included the schematic diagram of the 3D U-Net model in **Supplementary Figure 1**.

For kidney and mass segmentation, we employed two cascaded 3D U-Net models. First, the areas of the bilateral kidneys were determined on contrast-enhanced CT images employing a U-Net-based method. Then, the images were cropped according to segmented areas of the kidneys, and another U-Net network was trained for renal mass segmentation on the cropped images. The parameters of the segmentation model were as follows: filters=16, batch size=4, epochs=400, and learning rate=0.0001.

Additionally, we trained a 3D U-Net model (one-stage model) on the same dataset, with the same architecture and hyperparameters, to perform simultaneous segmentation of both the kidney and mass.

#### 2.6.2 Classification model training

Before training the classification model, the original images underwent a cropping process using the previous manual labels of each mass. The manual labels were overlaid onto the original images and the non-covered areas were automatically removed. Following this, the cropped images were resized to 128×128×128. Afterwards, we implemented several data augmentation techniques to expand the dataset, including randomly rotating the images by -10~10 degrees, introducing random noise, and randomly shifting the images horizontally and vertically -0.1~0.1.

The proposed classification method for renal masses was based on a 3D ResNet network (**Supplementary Figure 2**). The 3D ResNet architecture is designed to address the problem of vanishing gradients in very deep neural networks by using residual mappings instead of direct mappings (25). It consists of multiple residual blocks with shortcut connections that help the network achieve better accuracy while keeping the number of parameters relatively low.

To implement the proposed classification method, the contour of the mass was first extracted from the experimental dataset based on the labeling of experienced radiologists. Then, a 10-layer residual network was used, and the original network weights were preserved. A global average pooling layer, fully connected layer, and classification layer were constructed to complete the network. The network model was trained using the extracted experimental dataset with the following parameters: model depth=10, pretrained=1, hidden layer=128, dropout=0.1, batch size=4, epochs=400, and learning rate=0.0001. Finally, the ResNet-based method outputs the category with the highest probability.

To enhance the interpretability of our classification network, we generated class activation maps for the classification model, which highlight the regions of an image that are important for a particular classification decision. These maps provide insights into the reasoning of the network and help visualize the classification process. The class activation maps were generated using the Grad-CAM method, a popular technique that produces a heatmap indicating the contribution of each pixel in an input image to the classification decision (26).

### 2.7 Evaluation metrics

To assess the efficacy of our network in delineating kidney and renal masses from CT images, we compared the results of our algorithm’s segmentation to expert’s manual segmentation.

We utilized the Dice similarity coefficient (DSC) as a region-based metric to gauge the spatial overlap between the algorithm’s and expert’s segmentation. We also used the Hausdorff Distance (HD) as a boundary-based metric for evaluation.

**Figure 4:**
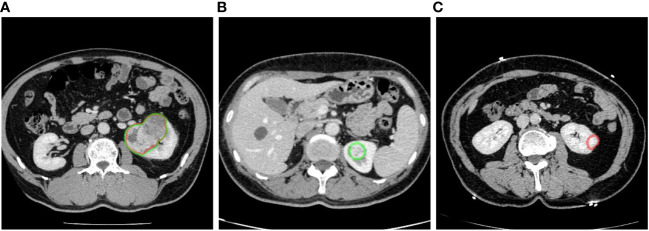
Examples of renal mass segmentation: **(A)** true positive, the predicted area of a renal mass (red) overlapped with the manual labeling area (green), **(B)** false negative, the predicted results missed the manual labeling area (green), and **(C)** false positive, there was a predicted area in the left kidney but it was not manually labeled. Red outline, segmented result; green outline, reference standard.

The detection efficacy of the segmentation models was evaluated at the connected domain level (**Figure 4**). For given connected domains with renal mass, if the algorithm prediction partially or fully overlapped the manual segmentation (reference standard), the prediction was considered true positive (TP), whereas if the domains were not included in the predicted region, the prediction was considered false negative (FN). For areas without renal mass, if the network predicted them as renal mass, it was counted as a false positive (FP).

For renal mass classification, we reported the accuracy, precision, recall and F1-score as evaluation metrics. Moreover, we used receiver operating characteristic (ROC) analysis and measured the area under the curve (AUC) value as the ability to distinguish between one specific type of renal mass and other renal masses on CT images. Studies have demonstrated that renal masses smaller than 10 mm, and in practice, those measuring 5 mm or less, are typically unable to be characterized on a CT scan (21). As a result, a threshold average diameter of 5 mm was used in the stratified analysis.

### 2.8 External validation

**Figure 5:**
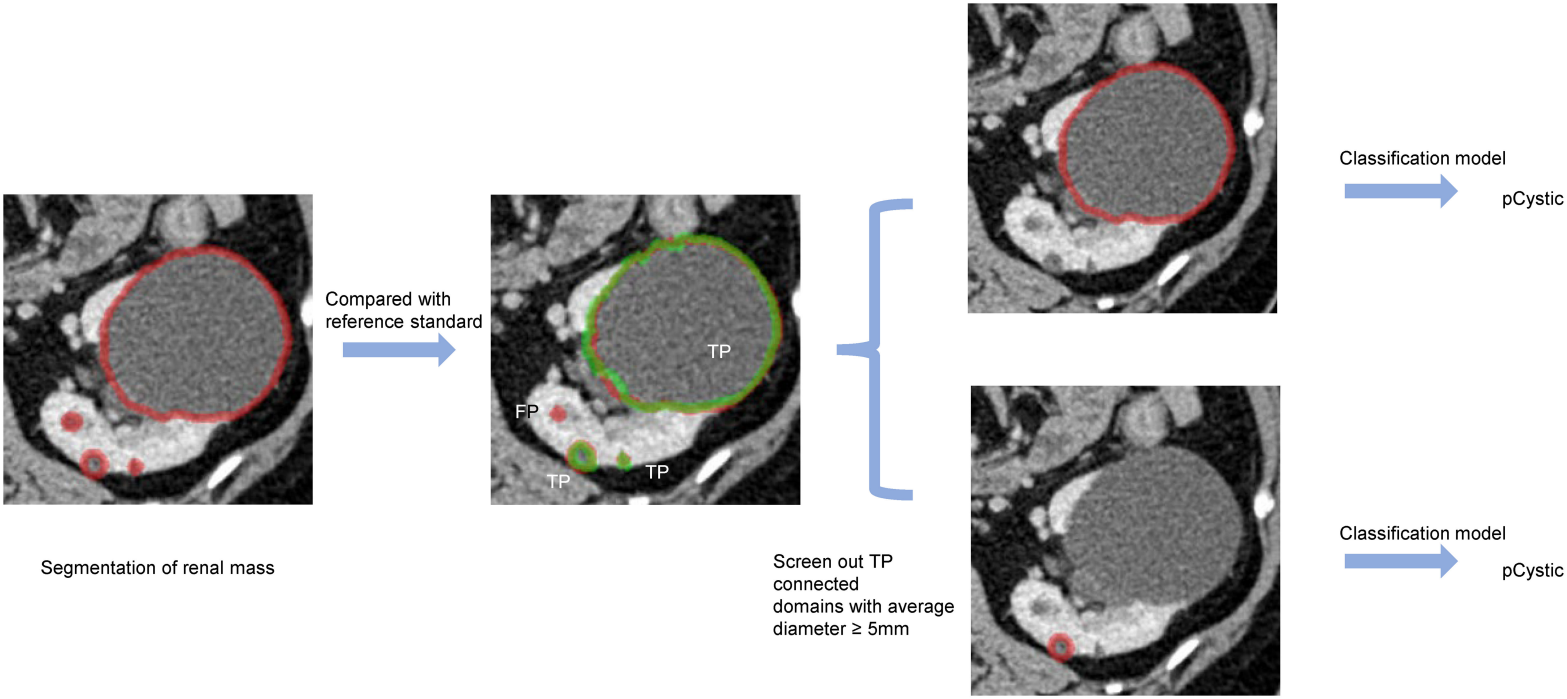
An example of predicted connected domains in the external validation set. Red outline, segmented result; green outline, reference standard.

In the external validation set, we employed a sequential approach using both the segmentation and classification models (**Figure 5**). First, we used the kidney and renal mass segmentation models to perform segmentation and compared the results with the reference standard. Then, the classification model was used to predict the most likely classification of the true positive predicted renal mass that had an average diameter greater than 5 mm, and these predictions were compared with the reference standard.

## 3 Results

### 3.1 Demographic characteristics

The kidney and renal mass dataset enrolled 490 patients during August 2009 and August 2021 (263 males and 227 females; mean age, 49.75 years ±18.72; range, 2-86 years). Corticomedullary phase (CMP) and nephrographic phase (NP) images were selected, and 610 image series were enrolled in total. Eighty-one patients between August 2018 and July 2022 were enrolled in the external validation dataset (42 males and 39 females; mean age, 51.01 years ±12.93; range, 23-77 years). There was no significant difference in gender and age between the two datasets (P>0.05).

All renal masses were confirmed by fellowship-trained radiologists for the analysis of cystic and solid renal masses, and the reference standard is described in section 2.3. We finally manually defined 198 AML, 1296 cystic masses and 397 solid masses in the 610 image series. These lesions were assigned into subgroups, which was consistent with kidney and renal mass segmentation above. In the external validation set, we manually defined 42 AML, 352 cystic masses and 33 solid masses in the 81 image series.

As some patients did not undergo surgery in our hospital, the histopathological information of some renal tumors was not collected. The detailed histopathological information of renal tumors is shown in **Supplementary Table 2**.

### 3.2 Result of the segmentation model

The results demonstrate that our algorithm is accurate for kidney segmentation with a mean DSC of 0.99 and 0.99 for the left and right kidneys in the test set, respectively (**Table 1**).

**Table 1:** Average DSC for kidney and renal mass segmentation of each dataset.

	Training set (n=487)	Validation set (n=58)	Test set (n=65)	KiTS21 dataset (n=300)	External validation set (n=81)
Left kidney	1.00	0.99	0.99	0.96	0.98
Right kidney	0.99	0.99	0.99	0.97	0.98
Left renal mass	0.91	0.78	0.75	0.68	0.70
Right renal mass	0.93	0.77	0.83	0.64	0.72

**Figure 6:**
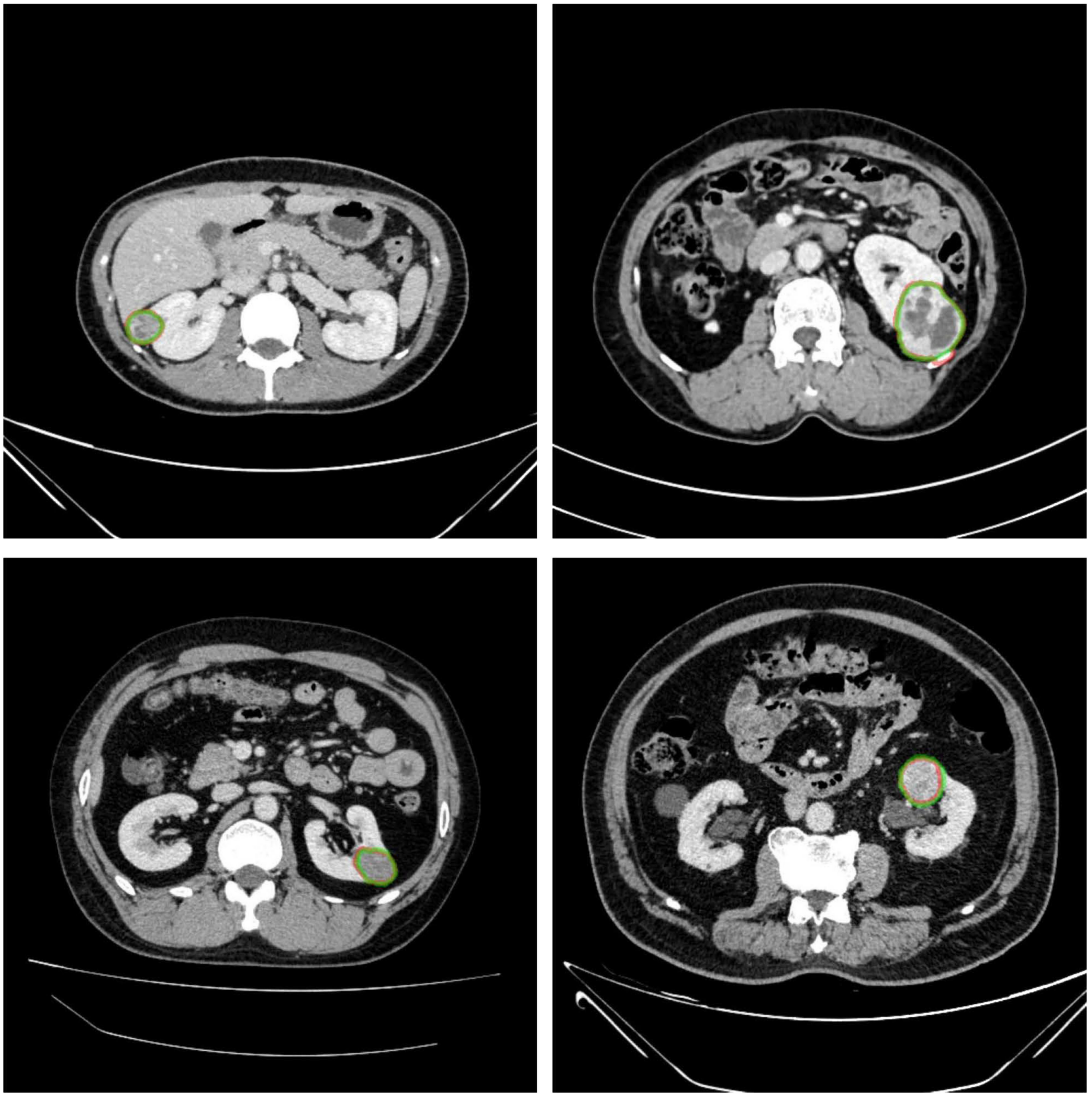
Example results of renal mass segmentation in four patients. Red outline, segmented result; green outline, reference standard.

**Figure 7:**
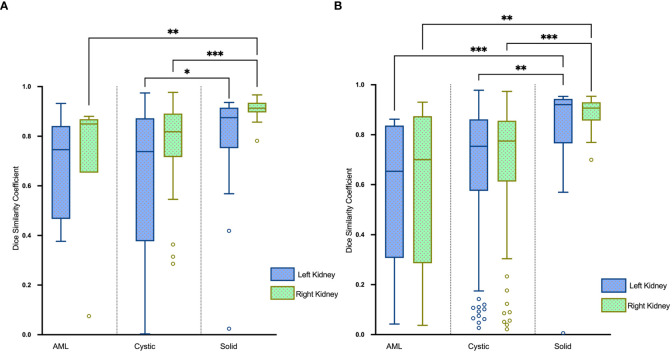
Boxplots of DSC for AML, cystic and solid renal mass segmentation of the **(A)** test set and **(B)** external validation set. *P<0.05, **P<0.01, ***P<0.001.

The renal mass segmentation model had a mean DSC of 0.75 for the left kidney and 0.83 for the right kidney in the test set (**Table 1**). Examples of segmentation results from the test datasets are given in **Figure 6**, where the algorithm-generated segmentation closely matches the manual segmentation. The algorithm performed better in solid renal mass segmentation compared to the other two types of masses for the right kidney (Kruskal-Wallis test and Dunn’s post test; P<0.01 and P<0.001), and for the left kidney, solid renal mass segmentation performed better than cystic mass segmentation (P<0.05), as shown in **Figure 7A**.

**Figure 8:**
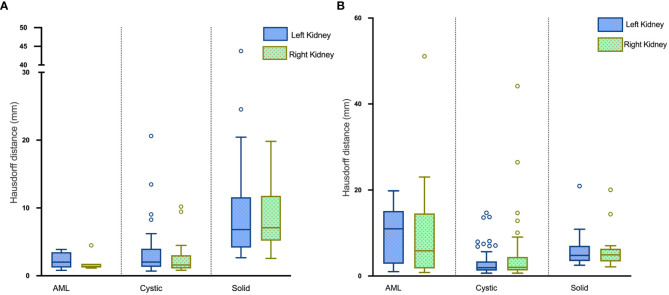
Boxplots of HDs for renal mass segmentation of the **(A)** test set and **(B)** external validation set.

The average HDs for segmentation of renal masses in the test set were 5.10 mm and 4.26 mm in the left and right kidneys, respectively. Boxplots of the results are visualized in **Figure 8A**.

In the test set, the precision in detecting renal masses in the left and right kidneys was 67.77% and 60.58% with corresponding recalls of 84.54% and 75.90%. The F1-scores were 0.75 and 0.67 (**Table 2**).

**Table 2:** Numbers of connected domains in renal mass segmentation of the test set and external validation set.

		True positive (TP)	False positive (FP)	False negative (FN)
Test set	Left	82	39	15
	Right	63	41	20
External validation set	Left	177	53	67
	Right	123	47	60

We evaluated the performance of our proposed kidney and renal mass segmentation models on the KiTS21 dataset, which comprises 300 CT scans with annotations for the kidney and renal masses. Our segmentation models achieved an overall DSC of 0.96 and 0.97 for kidney segmentation, and 0.68 and 0.64 for renal mass segmentation. It is worth noting that the performance of our model on the KiTS21 dataset was lower than on our local test set. This difference in performance may be due to disparities in data distribution and scan protocol between the two datasets, or to differences in the manual annotations of renal masses.

To further investigate the effectiveness of our proposed two-stage segmentation approach, we compared it with a one-stage model that was trained on the same dataset, using the same architecture and hyperparameters, to perform simultaneous segmentation of both the kidney and mass in **Supplementary Table 3**. The comparison results demonstrated that our two-stage segmentation approach outperformed the one-stage model in terms of segmentation on the test set.

### 3.3 Result of the classification model

**Figure 9:**
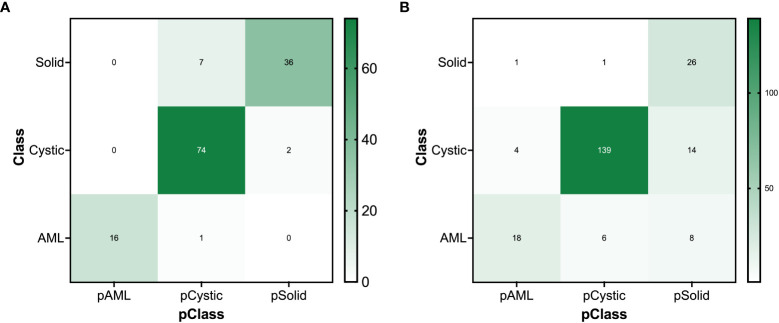
The confusion matrix for renal mass (average diameter≥5 mm) classification of the **(A)** test set and **(B)** external validation set.

**Figure 10:**
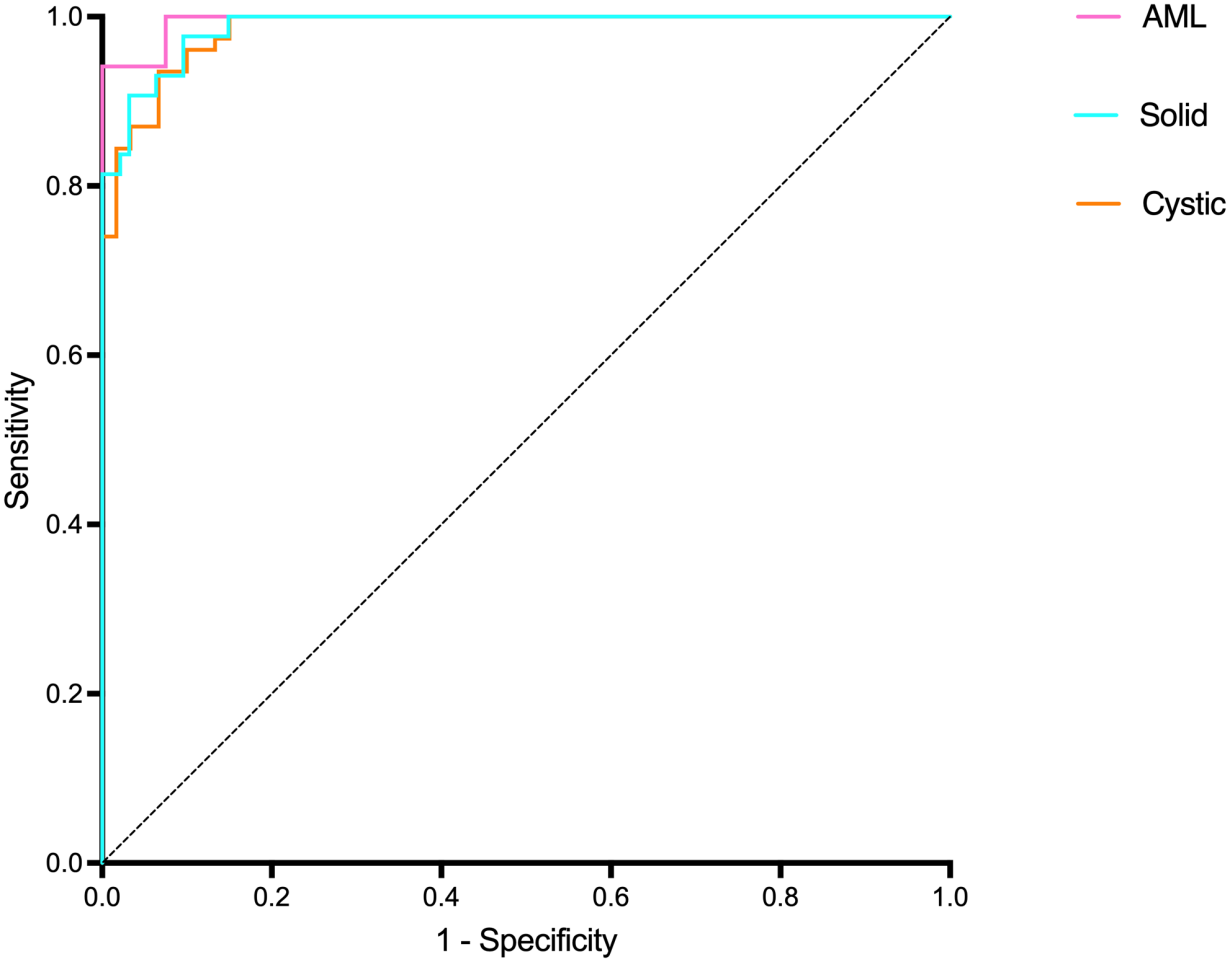
The ROC curves for renal mass (average diameter≥5 mm) classification in the test set.

**Table 3** and **Figure 9A** present the confusion matrix of the test set. In the test set, the accuracy was 90.56%. Furthermore, when the test set was divided into two groups based on lesion size, the accuracies were 86.05% for lesions smaller than 5 mm and 91.97% for lesions equal to or greater than 5 mm. Overall, the proposed method yielded higher accuracy in classification, especially in AML and cystic masses larger than 5 mm (**Table 4**). The AUCs for AML, cystic masses, and solid masses larger than 5 mm were 1.00, 0.98 and 0.99, respectively (**Figure 10**). Most solid masses misclassified in the test set were larger in size and had features of cystoid degeneration and necrosis. Additionally, most misclassified AML and cystic masses were smaller than 5 mm.

**Table 3:** Confusion matrix of the test set.

Average diameter (mm)		pAML	pCystic	pSolid	pUnknown
<5	AML	2	5	0	0
	Cystic	1	35	0	0
≥5	AML	16	1	0	0
	Cystic	0	74	2	1
	Solid	0	7	36	0

**Table 4:** Precision, recall and F score of the test set.

Average diameter (mm)		Precision	Recall	F score
<5	AML	66.67%	28.57%	0.40
	Cystic	87.50%	97.22%	0.92
≥5	AML	100.00%	94.12%	0.97
	Cystic	90.24%	96.10%	0.93
	Solid	94.74%	83.72%	0.89

To enhance the interpretability of our classification network, we generated class activation maps for the trained model, which highlight the regions of an image that are important for a particular classification decision. We present the class activation maps for several representative images in **Supplementary Figure 3**. As can be observed, the maps provide insight into the reasoning of the network and help visualize the classification process.

### 3.4 Results of the external validation set

#### 3.4.1 Kidney and renal mass segmentation

The mean DSC for kidney segmentation in the external validation set was 0.98.

The DSC for left renal mass was 0.70 and for right was 0.72 (**Table 1**). The algorithm performed better in solid renal mass segmentation in bilateral kidneys (Kruskal-Wallis test and Dunn’s post test; P<0.01 and P<0.001), as shown in **Figure 7B**. The average HDs for renal mass segmentation in the external validation set were 3.75 mm and 4.88 mm (**Figure 8B**). The precision, recall, and F1-score were 76.96%, 72.54%, and 0.76 for the left and 72.35%, 67.21%, and 0.70 for the right (**Table 2**).

Five solid masses had a DSC of 0, and one had a DSC of 0.006 in the external validation set, likely due to poor contrast with surrounding normal tissue resulting in nearly iso-density in NP images and even difficulty in visual observation.

#### 3.4.2 Renal mass classification

There were 300 TP domains and 100 FP domains after segmentation in our external validation set, and 217 TP connected domains had average diameters larger than 5 mm (**Figure 9B**). The proposed ResNet algorithm had a high accuracy of 84.33% and had a high sensitivity in identifying solid masses, as shown in **Table 5**.

**Table 5:** Precision, recall and F score of the external validation set.

	Precision	Recall	F score
AML	78.26%	56.25%	0.65
Cystic	95.21%	88.54%	0.92
Solid	54.17%	92.86%	0.68

Pearson correlation analysis also revealed a significant positive correlation (r=0.209, P<0.05) between the DSCs of TP renal mass connected domains larger than 5 mm and the accuracy of their classification predictions.

## 4 Discussion

This study presents a fully automated approach for detecting, segmenting, and classifying focal renal lesions in CT images. The detection of renal masses (cystic vs. solid, benign vs. malignant) is crucial in abdominal CT imaging for clinical diagnosis. Our method leverages a large institutional dataset of various types of renal masses obtained from multiple multidetector CT systems to achieve accurate detection. The performance of the proposed method was also evaluated using an external validation set and openly available KiTS21 dataset.

Our automated model demonstrated high accuracy in segmenting renal masses, achieving a high degree of spatial overlap with the reference standard, as demonstrated by the high DSC. This method requires no human intervention, making it a time-efficient and effective approach for evaluating renal masses. The ability to detect multiple lesions, a common occurrence in clinical practice, is another advantage of our model. These features enhance the ability to further classify renal masses into benign and malignant categories, providing valuable diagnostic information. However, our model has some limitations in segmenting cystic renal masses and AML, as shown by the lower DSC compared to solid masses. Additionally, poor contrast with surrounding normal tissue resulted in nearly iso-density in some images, leading to difficulty in visual observation and segmentation of some solid masses in NP images. Overall, our automated model provides an efficient and accurate approach for segmenting and classifying renal masses, with potential clinical applications.

The classification model showed high accuracy in distinguishing between different types of renal masses, with an overall accuracy of 90.56% in the test set. The proposed method also yielded higher accuracy in classification when lesions were larger than 5 mm. However, the model had difficulty in accurately classifying some solid masses with features of cystoid degeneration and necrosis. The use of class activation maps provided insight into the reasoning of the network and helped visualize the classification process. In the external validation set, the proposed ResNet algorithm had a high accuracy of 84.33% and had a high sensitivity in identifying solid masses. Overall, the classification model has the advantage of high accuracy and sensitivity, but has some limitations in accurately classifying certain types of solid masses.

Segmentation of renal masses is a crucial step in the development of computer-aided diagnostic and treatment planning tools. Two categories of previous methods exist: semi-automated and fully automated. Chen et al. used 3D segmentation software and interpolation to calculate the renal tumor volume in 27 patients with a high Lin’s concordance correlation coefficient (27). He et al. employed a grayscale adaptive network to simultaneously segment the kidney, renal tumors, arteries, and veins on CTA images in 123 patients, achieving an 86.4% DSC and 29.85 mm HD (28). Houshyar et al. utilized a CNN to segment the kidney and renal tumors from CT images in 319 patients, with median DSCs of 0.970 and 0.816 for kidney and tumor segmentation, respectively (29). Türk et al. employed a hybrid V-Net model to achieve an average DSC of 97.7% and 86.5% for kidney and tumor segmentation, respectively, on the KiTS19 dataset (30). They further enhanced both the encoder and decoder phases and incorporated a double-stage bottleneck block structure in the V-net model, resulting in a unique architecture that achieved an 86.9% DSC for kidney tumor segmentation (31). Compared to previous studies, our research used a larger dataset that covered most of the common renal mass types encountered in clinical practice. Although this may have led to a slightly lower segmentation performance compared to some of the studies mentioned above, we were able to achieve automatic segmentation and preliminary classification of renal masses, which is an important step towards developing computer-aided diagnostic and treatment planning tools for renal diseases. Furthermore, we validated our model on an external dataset, demonstrating its generalizability and potential clinical applicability. Overall, our study contributes to the growing body of literature on automated renal mass segmentation and classification, and provides a foundation for future research in this field.

Our study has some limitations to consider. First, it is retrospective in nature, and some of the renal masses did not have pathological information available. Imaging features, such as cystic or solid, should not be considered pathological features. Second, the impact of combining the segmentation and classification model has yet to be determined.

## 5 Conclusion

In conclusion, we developed a deep learning-based method for fully automated segmentation and classification of renal masses in CT images. Testing of this algorithm showed that it has the capability of accurately localizing and classifying renal masses.

## Data availability statement

The raw data supporting the conclusions of this article will be made available by the authors, without undue reservation.

## Ethics statement

The studies involving human participants were reviewed and approved by Committee for Medical Ethics, Peking University First Hospital. Written informed consent from the participants’ legal guardian/next of kin was not required to participate in this study in accordance with the national legislation and the institutional requirements.

## Author contributions

All authors contributed to the study conception and design. Material preparation, data collection and analysis were performed by TZ and ZS. TZ performed manual segmentation under the supervision of XW. ZS and YG participated in the image interpretation. YZ and YS performed data interpretation and statistical analysis. The first draft of the manuscript was written by TZ, and all authors commented on previous versions of the manuscript. All authors contributed to the article and approved the submitted version.

## References

[1] Kay FU Pedrosa I. Imaging of solid renal masses. Radiol Clin N Am (2017) 55:243–58. doi: 10.1016/j.rcl.2016.10.003 PMC5607740 28126214

[2] Hines JJ Eacobacci K Goyal R. The incidental renal mass- update on characterization and management. Radiol Clin N Am (2021) 59:631–46. doi: 10.1016/j.rcl.2021.03.011 34053610

[3] Silverman SG Pedrosa I Ellis JH Hindman NM Schieda N Smith AD. Bosniak classification of cystic renal masses, version 2019: an update proposal and needs assessment. Radiology (2019) 292:475–88. doi: 10.1148/radiol.2019182646 PMC6677285 31210616

[4] Hancock SB Georgiades CS Kidney Cancer. Cancer J (2016) 22:387–92. doi: 10.1097/PPO.0000000000000225 27870681

[5] Pierorazio PM Patel HD Johnson MH Sozio SM Sharma R Iyoha E. Distinguishing malignant and benign renal masses with composite models and nomograms: a systematic review and meta-analysis of clinically localized renal masses suspicious for malignancy. Cancer-Am Cancer Soc (2016) 122:3267–76. doi: 10.1002/cncr.30268 27508947

[6] Cohan RH Ellis JH. Renal masses: imaging evaluation. Radiol Clin N Am (2015) 53:985–1003. doi: 10.1016/j.rcl.2015.05.003 26321449

[7] Tang Z Yu D Ni T Zhao T Jin Y Dong E. Quantitative analysis of multiphase contrast-enhanced CT images: a pilot study of preoperative prediction of fat-poor angiomyolipoma and renal cell carcinoma. Am J Roentgenol (2020) 214:370–82. doi: 10.2214/AJR.19.21625 31799870

[8] Nie P Yang G Wang Z Yan L Miao W Hao D. A CT-based radiomics nomogram for differentiation of renal angiomyolipoma without visible fat from homogeneous clear cell renal cell carcinoma. Eur Radiol (2020) 30:1274–84. doi: 10.1007/s00330-019-06427-x 31506816

[9] Zhou L Zhang Z Chen YC Zhao ZY Yin XD Jiang HB. A deep learning-based radiomics model for differentiating benign and malignant renal tumors. Transl Oncol (2019) 12:292–300. doi: 10.1016/j.tranon.2018.10.012 30448734 PMC6299150

[10] Feng Z Rong P Cao P Zhou Q Zhu W Yan Z. Machine learning-based quantitative texture analysis of CT images of small renal masses: differentiation of angiomyolipoma without visible fat from renal cell carcinoma. Eur Radiol (2018) 28:1625–33. doi: 10.1007/s00330-017-5118-z 29134348

[11] Li ZC Zhai G Zhang J Wang Z Liu G Wu GY. Differentiation of clear cell and non-clear cell renal cell carcinomas by all-relevant radiomics features from multiphase CT: a VHL mutation perspective. Eur Radiol (2019) 29:3996–4007. doi: 10.1007/s00330-018-5872-6 30523454

[12] Ronneberger O Fischer P Brox T. U-Net: convolutional networks for biomedical image segmentation. Med Image Computing Computer-Assisted Intervention (2015), 234–41. doi: 10.1007/978-3-319-24574-4_28

[13] Norman B Pedoia V Majumdar S. Use of 2D U-net convolutional neural networks for automated cartilage and meniscus segmentation of knee MR imaging data to determine relaxometry and morphometry. Radiology (2018) 288:177–85. doi: 10.1148/radiol.2018172322 PMC6013406 29584598

[14] Man Y Huang Y Feng J Li X Wu F. Deep q learning driven CT pancreas segmentation with geometry-aware U-net. IEEE T Med Imaging (2019) 38:1971–80. doi: 10.1109/TMI.2019.2911588 30998461

[15] Nemoto T Futakami N Yagi M Kumabe A Takeda A Kunieda E. Efficacy evaluation of 2D, 3D U-net semantic segmentation and atlas-based segmentation of normal lungs excluding the trachea and main bronchi. J Radiat Res (2020) 61:257–64. doi: 10.1093/jrr/rrz086 PMC7246058 32043528

[16] Jiang D He J. Text semantic classification of long discourses based on neural networks with improved focal loss. Comput Intel Neurosc (2021) 2021:8845362. doi: 10.1155/2021/8845362 PMC7810536 33505454

[17] Ananda A Ngan KH Karabağ C Ter-Sarkisov A Alonso E Reyes-Aldasoro CC. Classification and visualisation of normal and abnormal radiographs; a comparison between eleven convolutional neural network architectures. Sensors (Basel Switzerland) (2021) 21:5381. doi: 10.3390/s21165381 34450821 PMC8400172

[18] Cejudo JE Chaurasia A Feldberg B Krois J Schwendicke F. Classification of dental radiographs using deep learning. J Clin Med (2021) 10:1496. doi: 10.3390/jcm10071496 33916800 PMC8038360

[19] Kokkalla S Kakarla J Venkateswarlu IB Singh M. Three-class brain tumor classification using deep dense inception residual network. Soft Comput (2021) 25:8721–9. doi: 10.1007/s00500-021-05748-8 PMC8051839 33897297

[20] Wang ZJ Westphalen AC Zagoria RJ. CT and MRI of small renal masses. Brit J Radiol (2018) 91:20180131. doi: 10.1259/bjr.20180131 29668296 PMC6221773

[21] Hélénon O Eiss D Debrito P Merran S Correas JM. How to characterise a solid renal mass: a new classification proposal for a simplified approach. Diagn Interv Imag (2012) 93:232–45. doi: 10.1016/j.diii.2012.01.016 22476035

[22] Sasaguri K Takahashi N. CT and MR imaging for solid renal mass characterization. Eur J Radiol (2018) 99:40–54. doi: 10.1016/j.ejrad.2017.12.008 29362150

[23] MICCAI. The 2021 kidney and kidney tumor segmentation challenge . Available at: https://kits-challenge.org/kits21 (Accessed 2021-7-1).

[24] Çiçek Ö Abdulkadir A Lienkamp SS Brox T Ronneberger O. 3D U-net: learning dense volumetric segmentation from sparse annotation, in: Ourselin S Joskowicz L Sabuncu MR Unal G Wells W editors. Medical Image Computing and Computer-Assisted Intervention–MICCAI 2016. Cham: Springer International Publishing (2016) 424–32. doi: 10.1007/978-3-319-46723-8_49

[25] He K Zhang X Ren S Sun J. Deep residual learning for image recognition, in: 2016 IEEE Conference on Computer Vision and Pattern Recognition (CVPR). Las Vegas, NV, USA: IEEE (2016) 770–8. doi: 10.1109/CVPR.2016.90

[26] Selvaraju RR Das A Vedantam R Cogswell M Parikh D Batra D. Grad-CAM: visual explanations from deep networks via gradient-based localization. Int J Comput Vision (2016) 128:336–59. doi: 10.1007/s11263-019-01228-7

[27] Chen MY Woodruff MA Kua B Rukin NJ. Rapid segmentation of renal tumours to calculate volume using 3D interpolation. J Digit Imaging (2021) 34:351–6. doi: 10.1007/s10278-020-00416-z PMC8289983 33564999

[28] He Y Yang G Yang J Ge R Kong Y Zhu X. Meta grayscale adaptive network for 3D integrated renal structures segmentation. Med Image Anal (2021) 71:102055. doi: 10.1016/j.media.2021.102055 33866259

[29] Houshyar R Glavis-Bloom J Bui T Chahine C Bardis MD Ushinsky A. Outcomes of artificial intelligence volumetric assessment of kidneys and renal tumors for preoperative assessment of nephron-sparing interventions. J Endourol (2021) 35:1411–8. doi: 10.1089/end.2020.1125 33847156

[30] Türk F Lüy M Barışçı N. Kidney and renal tumor segmentation using a hybrid V-Net-Based model. Mathematics (2020) 8:1772. doi: 10.3390/math8101772

[31] Turk F Luy M Barışçı N Yalçınkaya F. Kidney tumor segmentation using two-stage bottleneck block architecture. Intel Automation Soft Computing (2022) 33:349–63. doi: 10.32604/iasc.2022.023710

