# Supplementary material for: Automatic renal mass segmentation and classification on CT images based on 3D U-Net and ResNet algorithms
Source: Front Oncol. 2023 May 18;13:1169922. doi: 10.3389/fonc.2023.1169922 (PMC10233136; doi:10.3389/fonc.2023.1169922)
Supplement: Supplementary file 1 [file DataSheet_1.docx]

Supplementary Material

Automatic renal mass segmentation and classification on CT images based on 3D U-Net and ResNet algorithms

Tongtong Zhao^1^, Zhaonan Sun^1^, Ying Guo^1^, Yumeng Sun^2^, Yaofeng Zhang^2^, Xiaoying Wang^1^*

*** Correspondence:** Xiaoying Wang: wangxiaoying@bjmu.edu.cn

# Supplementary Figures and Tables

## Supplementary Figures


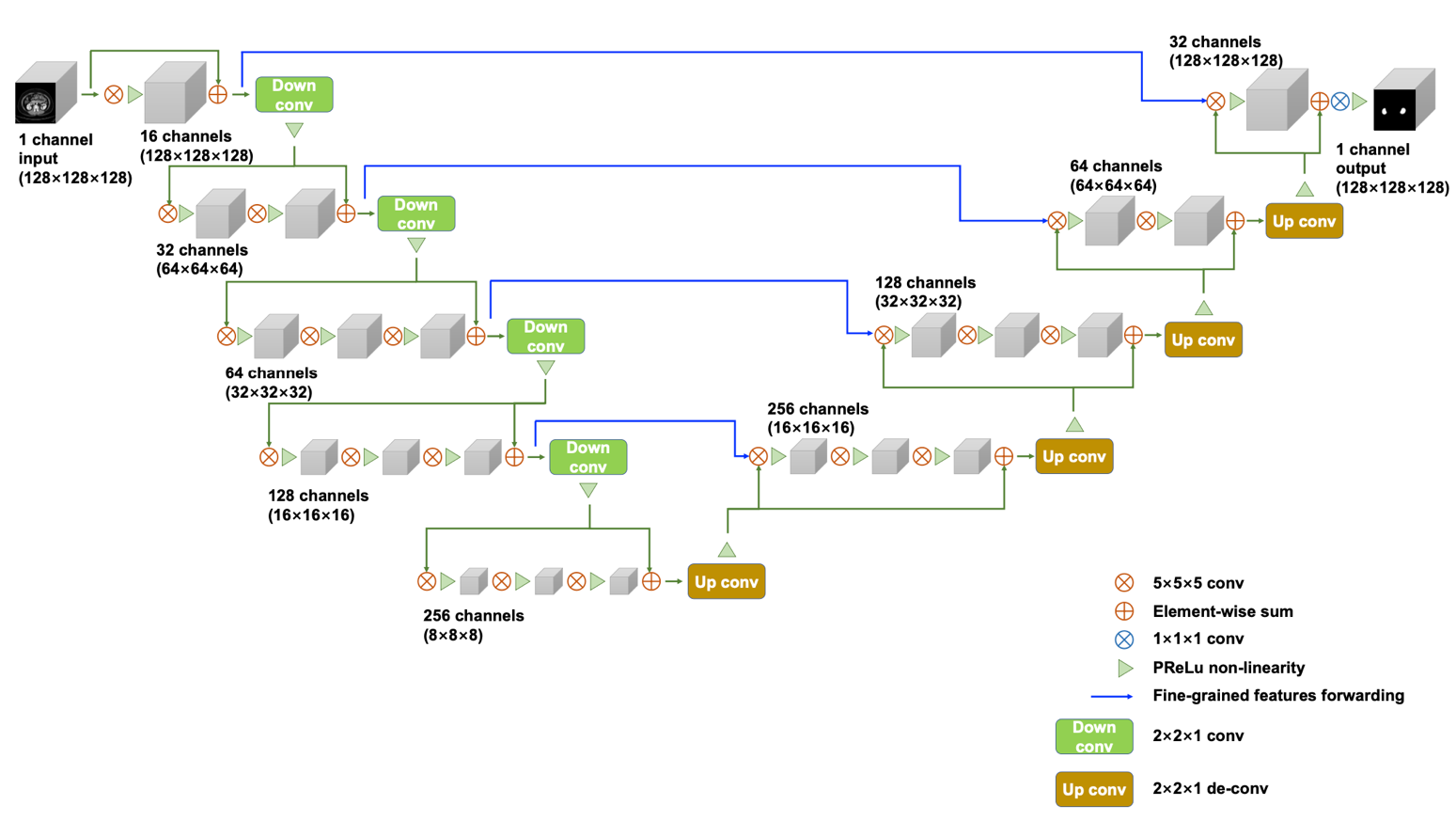


**Supplementary Figure 1.** Network structure of the 3D U-Net architecture. Conv, convolution; PReLu, Parametric rectified linear unit.


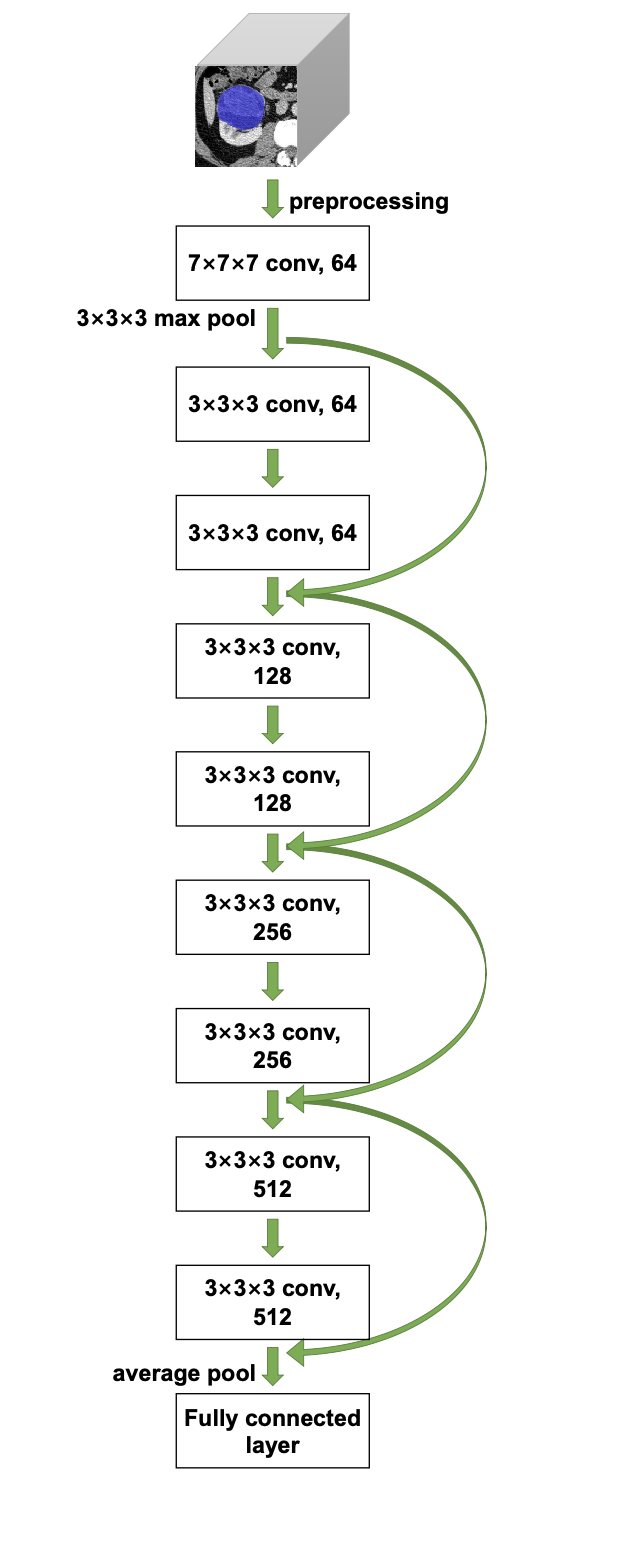


**Supplementary Figure 2.** Network structure of the 3D ResNet architecture. Conv, convolution.


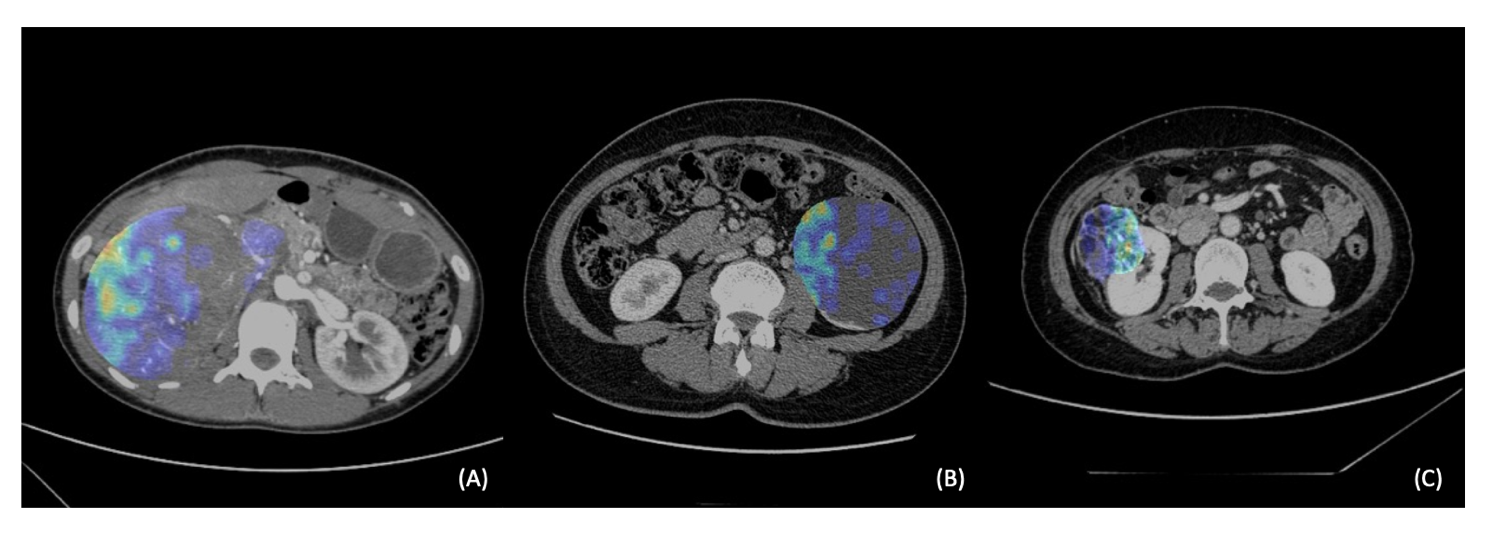


**Supplementary Figure 3.** Examples of class activation maps generated for (A) solid mass, (B) cystic mass and (C) AML. AML, angiomyolipoma.

## Supplementary Tables

**Supplementary Table 1.** Manufacture and model name in our hospital

| Manufacture | Manufacturer Model Name |
| --- | --- |
| GE Medical Systems | LightSpeed 16 |
|  | LightSpeed VCT |
|  | Discovery CT750 HD |
|  | Optima CT680 Expert |
| Neusoft | NeuViz Prime |
| Philips | Brilliance 64 |
|  | iCT 256 |
| SIEMENS | SOMATOM Definition Flash |

**Supplementary Table 2.** Histopathological information of renal tumors

| Class | Histopathological type | Training set | Validation set | Test set | External validation set | Total |
| --- | --- | --- | --- | --- | --- | --- |
| Cystic | Simple cyst | 26 | 3 | 3 | 4 | 36 |
|  | Cystic nephroma | 1 | - | - | 1 | 2 |
|  | Clear cell RCC | 2 | - | - | 1 | 3 |
|  | Chromophobe RCC | 1 | - | - | - | 1 |
|  | Unclassified RCC | 1 | - | - | - | 1 |
| Solid | Clear cell RCC | 147 | 22 | 18 | 24 | 211 |
|  | Papillary RCC | 23 | 3 | 1 | 1 | 28 |
|  | Chromophobe RCC | 30 | 6 | 3 | 4 | 43 |
|  | Xp11.2 translocation RCC | 1 | - | - | 2 | 3 |
|  | Clear cell papillary RCC | 1 | - | - | - | 1 |
|  | AML | 18 | 1 | 2 | 1 | 22 |
|  | Oncocytoma | 15 | - | 3 | - | 18 |
|  | Cystic nephroma | 1 | - | - | - | 1 |
|  | Inflammatory myofibroblastoma | 1 | - | - | - | 1 |
|  | Metanephric adenoma | 1 | - | - | - | 1 |
|  | Papillary adenoma | - | 1 | - | - | 1 |
|  | Urothelial carcinoma | 4 | 1 | 2 | - | 7 |
| AML | AML | 2 | - | - | 8 | 10 |
| Total |  | 275 | 37 | 32 | 46 | 390 |

RCC, renal cell carcinoma; AML, angiomyolipoma.

**Supplementary Table 3.** Average DSC for kidney and renal mass segmentation in one-stage model

|  | Training set (**n=487**) | Validation set  (**n=58**) | Test set  (**n=65**) |
| --- | --- | --- | --- |
| Left kidney | 0.98 | 0.98 | 0.97 |
| Right kidney | 0.98 | 0.98 | 0.98 |
| Left renal mass | 0.91 | 0.72 | 0.60 |
| Right renal mass | 0.88 | 0.65 | 0.70 |

**Supplementary Table 4.** Average DSC for kidney and renal mass segmentation model with batch 3 and 5

| Batch |  | Training set (**n=487**) | Validation set  (**n=58**) | Test set  (**n=65**) |
| --- | --- | --- | --- | --- |
| 3 | Left kidney | 0.99 | 0.99 | 0.99 |
|  | Right kidney | 0.99 | 0.99 | 0.99 |
|  | Left renal mass | 0.90 | 0.80 | 0.78 |
|  | Right renal mass | 0.94 | 0.84 | 0.76 |
| 5 | Left kidney | 0.99 | 0.99 | 0.99 |
|  | Right kidney | 0.99 | 0.99 | 0.99 |
|  | Left renal mass | 0.90 | 0.76 | 0.80 |
|  | Right renal mass | 0.91 | 0.82 | 0.68 |
